# Encephalitis due to herpes zoster without rash in an immunocompetent 12-year-old girl: case report and review of the literature

**DOI:** 10.1186/s12887-020-02244-0

**Published:** 2020-07-18

**Authors:** Silvia Ciancia, Antonella Crisafi, Ilaria Fontana, Alessandro De Fanti, Sergio Amarri, Lorenzo Iughetti

**Affiliations:** 1 Post-graduated School of Pediatrics, Department of Medical and Surgical Sciences for Mother, Children and Adults, University of Modena and Reggio Emilia, via del Pozzo 71, 41124 Modena, Italy; 2 Pediatrics Unit, Arcispedale Santa Maria Nuova, via Risorgimento 80, 42123 Reggio Emilia, Italy; 3 Pediatrics Unit, Department of Medical and Surgical Sciences for Mother, Children and Adults, University of Modena and Reggio Emilia, Via del Pozzo 71, 41124 Modena, Italy

**Keywords:** Varicella-zoster virus, VZV reactivation, Immunocompetent, Encephalitis, Meningitis

## Abstract

**Background:**

Neurological complications due to reactivation of varicella-zoster virus (VZV) are very uncommon in immunocompetent patients. Generally a vesicular rash is present on one or more dermatomes, preceding or following the main manifestation. Few cases are reported in the international literature, but they concern mainly adult or elderly patients.

**Case presentation:**

A 12-year-old girl was referred to our hospital for persisting headache, cough and rhinitis for six days. After first examination, diagnosis of anterior sinusitis was made by nasal endoscopy. The day after, the girl developed psychotic symptoms and altered mental status. Computed tomography (CT) scan was immediately performed but was unremarkable; lumbar puncture revealed leukocytosis with lymphocytic predominance and cerebrospinal fluid polymerase chain reaction (PCR) detected varicella-zoster virus DNA. The diagnosis of acute VZV encephalitis was made. The patient was promptly treated with acyclovir infused intravenously and her clinical conditions rapidly improved. Tests made did not show any condition of immunosuppression.

**Conclusions:**

Although if rare, reactivation of VZV can occur in immunocompetent children and its complications can involve central nervous system. Among these complications, meningitis is more common, but cerebral parenchyma can also be involved leading to a severe medical condition that is defined meningoencephalitis. In rare cases vesicular rash may be absent; therefore high level of suspicion is required even in those patients in which suggestive clinical features are not present to guide the diagnosis. Intravenous acyclovir represents the treatment of choice to obtain a fast clinical response and to prevent the onset of late-term complications.

## Background

Varicella-zoster virus (VZV) is a neurotrophic alphaherpesvirus infecting exclusively humans. VZV causes varicella (chickenpox) during the first infection (usually in childhood) and then becomes latent in cranial-nerve and dorsal-root ganglia. It can reactivate after several years causing zoster (shingles), sometimes followed by post-herpetic neuralgia. Reactivation generally occurs in elder population or in immunocompromised patients [1]. VZV can cause a wide spectrum of central nervous system (CNS) manifestations during first infection or later if reactivation occurs, such as encephalitis, cerebellitis, meningitis, vasculitis, stroke, polyneuropathy. The involvement of CNS can occur without concomitant skin eruption (zoster sine herpete) [2]. This scenario is uncommon both in children and in immunocompetent patients [3]. In this report, we describe a case of encephalitis due to VZV reactivation without the expression of vesicular rash in a 12-year-old immunocompetent girl.

## Case presentation

A 12-year-old girl was admitted to our hospital for persisting headache, started six days before. At the admission she presented also cough and rhinitis; she referred mild fever (axillary temperature 38 °C) during the previous days and three episodes of vomit. If at the beginning the pain was controlled by paracetamol, during the last days the headache had become deep, localized in the frontal region and associated to photophobia, even though at the beginning the pain was controlled by paracetamol. She had no personal or familial history of headache. Her medical history was unremarkable except for an episode of chickenpox during her infancy. On physical examination vital signs were stable (heart rate 90 bpm, blood pressure 111/73 mmHg, temperature 38 °C, GCS 15), the girl appeared suffering but her general conditions were good. Central nervous system examination didn’t show any neurological deficit, Kernings and Brudzinski signs were negative, she had no neck stiffness. Deep tendons reflexes were normal. Pupils were equal, round and reactive to light; accommodation reflex was present. Nor nystagmus or diplopia was noticed. Clinical examination of heart, lungs and abdomen was negative. On the skin no lesions were present. Blood count was in normal range, reactive C-protein (CRP) and procalcitonin (PCT) were negative, liver and renal functions were preserved. Analgesic and antipyretic treatment was administered. Nasal endoscopy was performed on the second day of hospitalization and highlighted a picture of acute bacterial sinusitis. Antibiotic therapy was started. The day after the girl developed altered mental status and psychomotor agitation; the headache worsened and was accompanied by three episodes of vomiting events; in few hours the girl developed drowsiness and became less responsive to stimuli. Blood tests were repeated but values were still in normal range. Computed tomography (CT) was performed immediately but was unremarkable. Electroencephalogram (EEG) showed “severe alteration of cortical electrogenesis with exacerbation of diffuse slow cortical activity, with fronto-temporal predominance”. Cerebrospinal fluid examination showed increased protein concentration (72 mg/dl), normal glucose concentration (52 mg/dl; blood glucose 105 mg/dl), lymphocytic pleocytosis (484 white cells/µL, 79% lymphocytes, 20% monocytes). The findings were suggestive for viral infection of CNS. Varicella-zoster virus was detected using polymerase chain reaction (PCR). The research of genome of herpes simplex virus 1 and 2, human herpes virus 6, cytomegalovirus and Ebstein-Barr virus was negative. Serological tests showed the presence of IgG vs. VZV and absence of IgM vs. VZV, confirming the reactivation of VZV. Immunological screening for HIV was negative, serum immunoglobulins, T and B lymphocytes counts, complement proteins levels were within normal range for age.

The girl was treated with intravenous acyclovir 10 mg/kg three times a day. During the first day of antiviral therapy her clinical conditions rapidly improved and the following day she was well oriented, smiling and quiet, not remembering the events of the days before. Her headache disappeared completely after two days. After 2 weeks of treatment, she was dismissed with oral acyclovir to be continued for 2 weeks more.

EEG was performed again after 3 and 12 days from the onset of encephalitis, showing progressive improvement of cortical activity, but a persistent slow focal activity in the right temporal region was registered. Magnetic resonance was performed after 2 days from the diagnosis of encephalitis and one month after the discharge and was unremarkable in both cases. Lumbar puncture was performed again after 14 days of therapy: protein concentration and glucose level were in normal range, a mild leukocytosis was still present (40 white cells/µL, with 93% of lymphocytes). PCR for VZV at that time was negative.

## Discussion and Conclusions

Varicella-zoster virus (VZV) causes two clinically distinct diseases. Varicella (or chickenpox) is the result of primary infection, it is extremely contagious and occurs mainly among preschool and school-aged children; the main characteristic is a generalized itchy vesicular rash. Although in most cases the evolution of varicella is benign, complications and atypical presentations can occur in previously healthy people, but their incidence is greater in immunocompromised patients [4]. After primary infection of varicella or after vaccination, VZV remains latent in the sensory ganglion cells and can reactivate if cell-mediated immunity declines as a consequence of aging of the patient or for the onset of an immunosuppressive state. Therefore, reactivation of VZV is rare in children [5].

It is known that varicella-zoster virus can cause several CNS disorders, such as meningitis, cerebellitis, myelitis, vasculitis and stroke-related syndromes. It was recognized as the second most common virus causing viral encephalitis, after herpes simplex virus. This frequency is declining in recent years in some countries after the introduction of VZV vaccination. VZV infections of the central nervous system are generally more frequent in immunocompromised individuals, but are not exclusive of this group of patients. In children, the most common CNS complication is cerebellitis that can develop during primary infection. In adults, encephalitis and meningitis have a higher incidence than in children and are usually subsequent to the reactivation of VZV [2]. In pediatric age encephalopathy develops almost exclusively after chickenpox and is related to Reye’s syndrome or to a process of perivenous inflammation and demyelination [1]. Neurological vascular complications are described both in adults and children [6]. Besides, pseudotumor cerebri has also been described as a rare neurological complication of VZV reactivation in pediatric age [7]. The reactivation of VZV in immunocompetent patients is uncommon, namely in childhood. Most of cases described in literature involve adults, both for encephalitis [8] and meningitis, more often associated with vesicular rash [9, 10].

The absence of vesicular rash preceding or following CNS involvement is rare. In a study of CNS complications of VZV carried in 84 children with neurological symptoms associated with VZV infection, only two patients had no rash, despite the diagnosis of VZV meningitis, confirmed by PCR analysis of the CFS [3].

The case we described presents peculiar medical features: encephalitis was caused by reactivation of VZV in an immunocompetent child, who did not present the typical rash. Consequently, we decided to perform a systematic review of the literature on PubMed database matching words “encephalitis”, “central nervous system”, “immunocompetent”, and “varicella zoster”. Of 103 results obtained, we analyzed title and abstract of all relevant papers, regardless of language. We found only two reported cases of encephalitis due to VZV reactivation in pediatric patients in absence of waning of immune system function. A comparison between our case and previous described ones is showed in Table 1 [11, 12]. Patient described by Chiappini et al. had a younger age and the authors presume that early primary infection by VZV could be a risk factor for subsequent reactivation and CNS manifestations. The case we described and the case reported by Spiegel et al. do not support this hypothesis. From the analysis of mentioned cases, previous VZV reactivations do not seem to represent a risk factor. According to the cerebrospinal fluid findings, in our patient and in Spiegel et al. patient the involvement of meninges support the diagnosis of meningoencephalitis. EEG was abnormal in all the three patients, proving the involvement of cerebral parenchyma; neuroimaging in our patient was unremarkable. Even if meningitis due to VZV is more common after a primary infection, some cases of meningitis due to VZV reactivation in immunocompetent children have been reported, mainly (but not exclusively) associated to the presence of vesicular rash [13–15]. In all these cases any involvement of cerebral parenchyma was demonstrated and this supports one of unique feature of our case. To our knowledge, some cases of CNS infection due to reactivation of VZV in vaccinated children have been reported, but genotyping verification of the vaccine strain was available in a relatively small number of patients [16–18]. Regardless our patient, as the other two showed in the table, presented varicella during their infancy and they did not get vaccination.

In our opinion, it is significant to highlight that none of the three patients discussed showed the vesicular rash, therefore we should keep a high level of suspicion of VZV infection of central nervous system even in the absence of suggestive diagnostic clinical features. 

In the suspicion of VZV encephalitis, polymerase chain reaction (PCR) allows the detection of viral DNA and RNA in CSF samples with high sensitivity and specificity [19, 20]. Because virus isolation and detection of intrathecal antibody production usually need at least 10 days after the onset of symptoms to be achieved, PCR is the best technique to make a prompt diagnosis and to start the correct treatment. Moreover herpesvirus isolation from early CSF is exceptional [21]. In our patient, positive serum IgG and negative IgM allow to distinguish a primary infection from reactivation.

Intravenous acyclovir 10–15 mg/kg every 8 h is the treatment of choice; the recommended duration is 14 days but if compromised immune system function is suspected or known, the treatment should be extended to 21 days. Many experts recommend adjunct corticosteroids if CNS vasculitis is present [22].

Even if rare, reactivation of VZV with subsequent involvement of central nervous system can occur in previous healthy people, including children. Vesicular rash could be absent; therefore high level of suspicion for VZV infection has to be kept in patients with clinical, biochemical and instrumental findings suggestive for viral meningoencephalitis. PCR of cerebrospinal fluid is the faster tool to achieve a correct diagnosis and start a prompt therapy. Intravenous acyclovir is the drug of choice.

**Table 1:** Reported cases of encephalitis caused by varicella zoster virus reactivation in immunocompetent children

	Chiappini et al., 2002 [11]	Spiegel et al., 2010 [12]	Our patient, 2020
Age and gender	2-year-old boy	14-year-old girl	12-year-old girl
Chickenpox/vaccination (age)	Chickenpox (4 months)	Chickenpox (4 years)	Chickenpox (NA)
Previous reactivation	No	Zoster (10 years)	No
Clinical presentation:			
-Fever	Yes (T 38.5 °C)	Yes (T 39.2 °C)	Yes (T 38 °C)
-Neurological symptoms & signs	Frontal headache, vomiting, disturbed consciousness, miotic pupils, tendon reflexes absent, left ankle clonus, bilateral Babinski’s reflexes elicited	Paresthesias and hyperesthesia at left limbs, proximal left leg weakness; severe frontal and occipital headache, stiff neck, positive Brudjinski sign	Severe frontal headache, vomiting, photophobia, altered mental status, psychomotor agitation.
- Skin rash	No	No	No
CSF:			
- white cells/mL	Normal	434	484
- protein (mg/dL)	Normal	59	72
- glucose (mg/dL)	Normal	49	52
- PCR for VZV-DNA	Positive	Positive	Positive
IgG and IgM VZV	Serum: IgG +, IgM - CSF: IgG and IgM -	Serum: IgG +, IgM -	Serum: IgG +, IgM -
EEG	Findings compatible with viral encephalitis	Right-hand sided slow waves, compatible with right hemisphere encephalopathy	Severe alteration of cortical electrogenesis with exacerbation of diffuse slow cortical activity, with fronto-temporal predominance
MRI	Bilateral multifocal changes in white and gray matter, predominantly on the fronto–parietal cortex	4 hyperintense lesions without enhancement after gadolinium injection: 1 in right thalamus, 1 in right temporal subcortical region, 2 in right parietal subcortical region	Unremarkable
Immunological screening	HIV Ab negative; IgG, IgA, IgM serum levels, B and T-lymphocyte counts, NK cell subset counts, in vitro T-lymphocyte response to mitogens, MHC I and II class molecule-positive cell counts: normal.	HIV Ab negative; IgG, IgA, IgM serum levels, complement studies, total counts and functional studies of T cells: normal.	HIV Ab negative; IgG, IgA, IgM serum levels, B and T-lymphocyte counts
Treatment (days)	Acyclovir iv (15 days); ceftriaxone iv (5 days)	Acyclovir iv (14 days); methylprednisolone iv (5 days)	Acyclovir iv (14 days) followed by oral acyclovir (14 days)
Sequelae	Mild right hemiparesis	Left thigh neuralgia	No

## Data Availability

Data sharing is not applicable to this article as no datasets were generated or analyzed during the current study.

## Abbreviations

CNS: Central nervous system
CRP: C-reactive protein
CSF: Cerebrospinal fluid
CT: Computed tomography
EEG: Electroencephalography
IgG: Immunoglobulin G
IgM: Immunoglobulin M
PCR: Polymerase chain reaction
PCT: Procalcitonin
VZV: Varicella zoster virus
